# Interpersonal violence and mental health: a social justice framework to advance research and practice

**DOI:** 10.1017/gmh.2020.4

**Published:** 2020-05-06

**Authors:** W.A. Tol

**Affiliations:** 1 Department of Mental Health, Johns Hopkins Bloomberg School of Public Health, Baltimore, Maryland, USA; 2 Peter C. Alderman Program for Global Mental Health, HealthRight International, New York, NY, USA

**Keywords:** Global mental health, interpersonal violence, mental health, social justice

## Abstract

This editorial paper accompanies a special series in the journal Global Mental Health focused on the topic of interpersonal violence and mental health. This series included 24 papers reporting on data from 31 countries, published between 2017 and 2019. This accompanying paper provides a short summary of findings in the special series and reflects on next steps in research and practice. Collectively, the series’ 24 papers suggest intricate bi-directional relationships between interpersonal violence and mental health, situated in particular contexts and varying across the life course. In order to study this complexity, an overarching theoretical framework is critical. This paper takes the social justice theory developed by Powers and Faden (2006, 2019) as a starting point. It is argued that application of this social justice framework will be helpful to: strengthen conceptual clarity; provide a sense of direction for research and practice in the area of interpersonal violence and mental health; assist in conducting more fine grained analyses of contextually determined processes of disadvantage; and help situate disciplinary specific research and practice questions in their broader context, thereby strengthening multi-disciplinary research and multi-sectoral policy and programming efforts.

In 2017, this journal published a call for papers for a special series on the topic of interpersonal violence and mental health. The invitation was received with much interest: from 2017 to 2019, 24 papers were published reporting on data from 31 countries. In this editorial paper my aims are to provide a short background to the series; provide a succinct overview of its content; and reflect on next steps. In doing so, I will draw on a social justice theoretical framework developed by Powers and Faden (2006), (2019). Powers’ and Faden's work makes the case for social justice as the moral foundation for public health and policy, with human wellbeing as the ultimate aim that principles of justice aim to achieve. The theoretical framework's emphasis on identifying interconnected patterns of disadvantage provides a good fit with emerging research findings indicating that interpersonal violence and mental health are linked in complex bi-directional relationships (e.g. vicious cycles). Applying this social justice lens, the editorial paper will focus particularly on the kind of research that is required to break the systematic, interlocking, patterns of disadvantage that tie interpersonal violence and mental health.

## Defining interpersonal violence

Violence is a ‘slippery concept’ (Scheper-Hughes and Bourgois, 2004), but is often characterized with regard to two elements: (1) intentionality (at least partly), and (2) infliction of damage (Achterhuis, 2008). In its World Report on Violence and Health, the World Health Organization (WHO) defines violence as ‘the intentional use of physical force or power, threatened or actual, against oneself, another person, or against a group or community, that either results in or has a high likelihood of resulting in injury, death, psychological harm, maldevelopment or deprivation’ (Dahlberg and Krug, 2002). In turn, the 2002 WHO report distinguishes interpersonal violence, the topic of this special issue, from self-directed violence and collective violence. What characterizes interpersonal violence is that it is committed by individuals, or small groups of individuals, against other individuals or small groups of individuals – as opposed to by a person against themselves (self-directed violence) or by larger groups such as states, militia or other armed groups (collective violence). Violence can be physical, sexual, and psychological in nature, as well as involve deprivation and neglect. Examples of interpersonal violence include intimate partner violence (IPV), child abuse and neglect, random acts of violence, sexual assault by strangers, violence committed by teachers against pupils, and communal violence (Dahlberg and Krug, 2002).

## Scope and consequences

Interpersonal violence is highly prevalent and affects both men and women, although perpetrators are more commonly men (World Health Organization, 2017). One of the most common forms of interpersonal violence concerns IPV, which is reported to have occurred in their lifetimes by on average 1 out of 3 women (30.0%) aged 15 years and older, according to a synthesis of data from 141 studies in 81 countries (Devries *et al*., 2013*b*). Sexual violence by non-partners (commonly people known to the victim) has an average prevalence of 7.2% globally in women (Abrahams *et al*., 2014). Sexual violence against men has not been well documented in low- and middle-income countries, but has been estimated at 6.0% in the US (compared to 14.7% in the same study in women) (Mitra *et al*., 2016). Child abuse and neglect affects millions of children globally, with average lifetime prevalence rates of 12.7% for sexual abuse (7.6% boys, 18.0% girls); 22.6% for physical abuse; 36.3% for emotional abuse; 16.3% for physical neglect; and 18.4% for emotional neglect (Stoltenborgh *et al*., 2015). Homicide caused the death of 0.5 million people in 2012 (United Nations Office on Drugs and Crime, 2014), with 13.5% of homicides committed by intimate partners, mostly male perpetrators and female victims (Stockl *et al*., 2013). Rates of different types of interpersonal violence vary widely across world regions (e.g. for IPV from 19.3 to 65.6%) (Devries *et al*., 2013*b*).

The health consequences and economic costs of interpersonal violence are staggering. For survivors, interpersonal violence has important physical and mental health consequences (Ellsberg *et al*., 2008), a focus of multiple studies included in this series. With regard to economic impacts, interpersonal violence costs the USA 3.3% of its gross domestic product. There are fewer studies from low- and middle-income countries, but IPV for example costs 1.6% of gross domestic product in Nicaragua, and 2.0% in Chile (Waters *et al*., 2004).

## Interpersonal violence and mental health: overview of the series

**Fig. 1.**
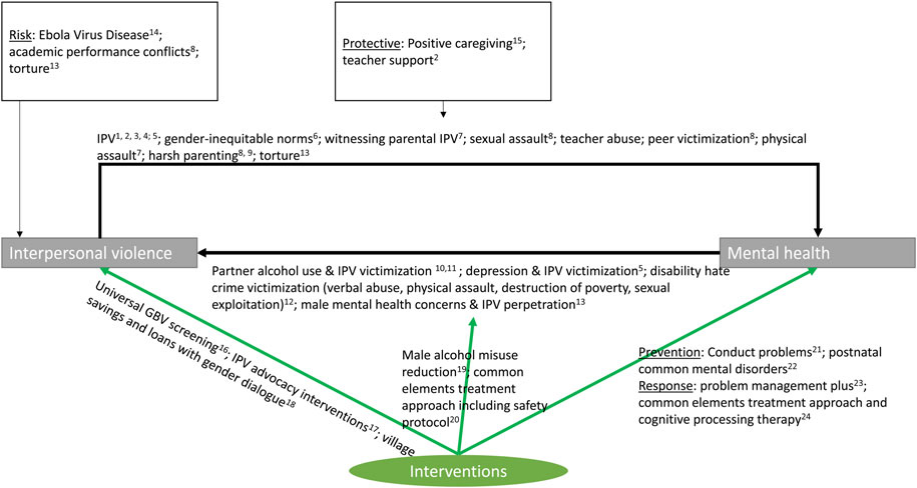
Overview of findings in the special series.

^1^Bajunirwe *et al*. (2018); ^2^Bhardwaj *et al*. (2018); ^3^Glass *et al*. (2018); ^4^Malan *et al*. (2018); ^5^Schneider *et al*. (2018); ^6^Stark *et al*. (2018); ^7^Meyer *et al*. (2017); ^8^Le *et al*. (2018); ^9^Baker-Henningham & Francis (2018); ^10^Greene *et al*. (2017); ^11^Schafer and Koyiet (2018); ^12^Carr *et al*. (2017); ^13^Rees *et al*. (2018); ^14^Green *et al*. (2018); ^15^Berckmoes *et al*. (2017); ^16^Vu *et al*. (2017); ^17^Tiwari *et al*. (2018); ^18^Annan *et al*. (2017); ^19^Giusto and Puffer (2018); ^20^Kane *et al*. (2017); ^21^Baker-Henningham & Walker (2018); ^22^Fisher *et al*. (2018); ^23^Van ‘t Hof *et al*. (2018); ^24^Mahmooth *et al*. (2018).

Figure 1 aims to provide a schematic overview of findings in the series. The majority of studies have focused on mental health as the main outcome of interest, either studying the impacts of interpersonal violence on mental health, or examining the mental health benefits of psychosocial interventions with violence-affected populations. At the same time, the reverse relationship (i.e. from mental health to interpersonal violence) has received increasing attention in recent years (Tol *et al*., 2019) – and is the focus of a number of papers in this series as well. Below, I first highlight etiological findings of the studies that have aimed to identify pathways between interpersonal violence and mental health (in both directions), and subsequently discuss the intervention-focused studies.

### Pathways between interpersonal violence and mental health

#### Interpersonal violence as a risk factor for mental health

A commonly cited limitation in the literature on interpersonal violence as a risk factor for mental health is the use of cross-sectional designs (Cerda *et al*., 2012). However, five of the 10 studies that have had this focus in the series were longitudinal in nature. Two longitudinal studies have examined harsh parenting. In Liberia, a longitudinal study (*n* = 185) found that parents from households with reported Ebola virus disease sickness became harsher parents over a 12-month period compared to non-exposed parents (Green *et al*., 2018). In a longitudinal study amongst families in Jamaica, a higher frequency of harsh parenting in the pre-school period was associated with increased problem behaviors for children in grade 1 (*n* = 211) (Baker-Henningham and Francis, 2018). Of note, a qualitative (non-longitudinal) study with 74 armed conflict-affected households in Burundi conversely indicated that positive caregiving was congruent with patterns of resilience (Berckmoes *et al*., 2017).

Several studies have looked at IPV as a particular form of interpersonal violence – covering various stages across the life course. A longitudinal study in Nepal with former child soldiers and matched civilian children (*n* = 290) (on average 16 years old at the time of recruitment) found that exposure to sexual IPV (18% in women, 7% in men) was a predictor for suicidal attempts 5 years later (Bhardwaj *et al*., 2018). In a longitudinal study amongst perinatal women in informal settlements in South Africa (*n* = 425), IPV victimization at baseline was associated with higher levels of psychological distress, food insecurity, and rates of alcohol misuse (Schneider *et al*., 2018). A longitudinal study in armed conflict-affected eastern Democratic Republic of the Congo (*n* = 380) found mental health impacts of IPV and parental mental health concerns on adolescents (Glass *et al*., 2018).

These longitudinal and several cross-sectional (Meyer *et al*., 2017; Bajunirwe *et al*., 2018; Malan *et al*., 2018; Rees *et al*., 2018) and qualitative studies (Le *et al*., 2018) in the series confirm the negative impacts from various types of interpersonal violence on a range of mental health outcomes, including suicidality.

#### Mental health as a risk factor for interpersonal violence

Regarding the inverse relationship from mental health to interpersonal violence, several studies have examined how mental health concerns in violence-affected populations may be associated with the perpetration of violence or (further) victimization. Concerning the perpetration of interpersonal violence, findings in this series point to an important pathway between male alcohol misuse and perpetration of IPV (Greene *et al*., 2017; Schafer and Koyiet, 2018). A study in post-conflict Timor-Leste (*n* = 870) identified a pathway where men who had been tortured experienced a range of mental health concerns (posttraumatic stress and depression symptoms, alcohol misuse), which increased the chances that their partners reported IPV (Rees *et al*., 2018). With regard to interpersonal violence victimization, the aforementioned longitudinal study with South African perinatal women found a higher incidence of IPV in women with depression at baseline (Schneider *et al*., 2018). Furthermore, a scoping review summarized 13 studies that have looked at experiences of violence and hostility against mental health service users (‘disability hate crime’) in the United Kingdom (Carr *et al*., 2017). The scoping review's authors particularly emphasize the lack of service user-led efforts in both intervention and research.

Together with other recent studies (Perez and Johnson, 2008; Devries *et al*., 2013*a*; Kim and Lee, 2013; Tsai *et al*., 2016), the above findings are starting to demonstrate complex bi-directional relationships between violence and mental health.

### Intervention research

#### Preventing and reducing adverse mental health outcomes

To address the documented negative impacts of interpersonal violence victimization on mental health, a growing number of studies have rigorously evaluated interventions that may prevent or reduce mental health concerns in violence-affected populations in low-resource settings (green arrow at the bottom right, Fig. 1). In this series, two randomized controlled trials (RCTs) have examined whether interventions can successfully prevent adverse mental health outcomes. In Jamaica, an 8-day training program was successful in improving teacher's skills to manage classrooms and also found reduced problem behaviors and increased educational engagement in children (*n* = 225) (Baker-Henningham and Walker, 2018). In Australia, a universal perinatal prevention intervention with first-time mothers (*n* = 314) aimed to improve relationships with an intimate partner and management of unsettled infant behaviors. Follow-up findings showed lower anxiety scores at 12-months compared to the control condition (Fisher *et al*., 2018).

In addition to prevention, several papers report on efforts to reduce mental health concerns once arisen. In Iraq, a RCT with 342 participants examined the effectiveness of two cognitive behavioral interventions in reducing locally prioritized mental health complaints, and found that a treatment combining multiple evidence-based elements (Common Elements Treatment Approach, CETA) was effective in doing this, with few effects for cognitive processing therapy (Mahmooth *et al*., 2018). Separately, a post-RCT qualitative process evaluation of a different transdiagnostic intervention developed by the WHO found that stakeholders in informal settlements in Kenya largely found the intervention feasible and acceptable for this context (Van't Hof *et al*., 2018).

#### Reducing interpersonal violence

Other studies in the series have focused on interpersonal violence as the main outcome, with improvements in mental health as the hypothesized intervention ingredient to achieve a reduction in interpersonal violence (green arrow in the middle, Fig. 1). Giusto and Puffer (2018) conducted a systematic review to identify evaluations of interventions focused on male alcohol misuse, to understand the impact of these interventions on family outcomes, including IPV. Overall, a scarcity of interventions have studied this topic rigorously (Giusto and Puffer, 2018). Given the current lack of studies, the RCT protocol aiming to understand the impacts of CETA with couples in Zambia on violence against women and girls in families is a welcome effort to improve knowledge in this area (Kane *et al*., 2017).

In addition, studies have examined whether interventions may reduce interpersonal violence via other non-mental health mechanisms (green arrow to the left, Fig. 1). Vu and colleagues found that a universal screening approach integrated in refugee health clinics was broadly considered feasible and acceptable, but that finding private space for screening was challenging (Vu *et al*., 2017). In addition, two papers have specifically looked at whether an interpersonal violence-focused intervention may have benefits for mental health. In Cote d'Ivoire, a pilot RCT found that adding a couples gender discussion group to an economic group savings intervention (village savings and loans) was associated with a larger reduction in posttraumatic stress disorder symptoms in women, but only for women without a history of IPV (Annan *et al*., 2017). Tiwari *et al*. (2018) provide a useful comment on research from four empowerment-based advocacy interventions with Chinese women survivors of IPV. RCTs of these interventions have found benefits for depressive symptoms (Tiwari *et al*., 2018).

## A social justice framework

The image that emerges from the findings across the series’ 24 papers is one of vicious cycles in which interpersonal violence and mental health reinforce each other, rather than simple unidirectional relationships from interpersonal violence as a risk factor to mental health symptoms as the outcome. The notion of vicious cycles appears to be relevant for other critical social issues with relevance to global mental health such as poverty (Lund *et al*., 2011, 2018). To further add to the complexity, interpersonal violence and mental health themselves do not occur in a vacuum, but are driven by broader contextual processes. In this series, for example, infectious disease epidemics (Green *et al*., 2018) and political violence (Rees *et al*., 2018) were found to increase risks for interpersonal violence. In order to study this complexity, while still seeing the forest for the trees, an overarching theoretical framework will be critical. I argue here that a theoretical framework focused on social justice provides a helpful starting point.

In their 2006 book, bioethicists Powers and Faden put forward a theory of social justice to facilitate an analysis of the moral issues at stake in public health and health policy questions. They have since expanded the theory in their most recent book (2019). Their work makes the case for social justice as the moral foundation for public health, in contrast to the more usual considerations of bringing about the greatest health benefits from limited public health resources. Powers’ and Faden's theory is posited as a ‘non-ideal’ theory, in the sense that it provides a framework for analyzing which inequalities matter most in the real world, where many millions have unmet basic needs and no secure liberties and human rights violations are commonplace. Their starting point is a consideration of the ultimate purpose that principles of justice aim to achieve, which they consider to be human wellbeing (Powers and Faden, 2006, 2019).

**Fig. 2.**
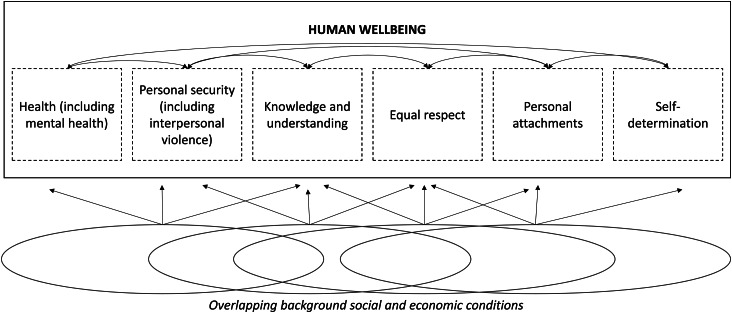
Overlapping background social and economic conditions.

Wellbeing is subsequently described as consisting of six irreducible elements, each representing something of independent moral significance. These elements are: health (including mental health), personal security (encompassing freedom from interpersonal violence as defined above, but also other forms of violence), knowledge and understanding, equal respect, personal attachments, and self-determination (Fig. 2). A life significantly lacking in one of these dimensions, Powers and Faden argue, is a life seriously deficient in what is reasonable for anyone to want, whatever else they want. The ‘job’ of social justice then is ‘to specify those background social and economic conditions that determine whether certain inequalities, that may themselves result from the promotion of other indispensable moral aims, should be seen as unfair’ (Powers and Faden, 2006).

This specification of the job of social justice aligns with the social determinants tradition in public (mental) health [e.g. (Allen *et al*., 2014; Tol, 2015; Lund *et al*., 2018)] and the emerging literature on syndemics and interpersonal violence (Mendenhall, 2015; National Academies of Sciences and Medicine, 2019). At the same time, this non-ideal social justice framework asks us to consider, more broadly, how determinants of health function alongside determinants of other elements of wellbeing. The framework also asks us to consider how the six wellbeing elements inter-relate (see Fig. 2). Central to the social justice perspective put forward by Powers and Faden is the assertion that the greatest moral urgency should be accorded to populations who fare badly on multiple dimensions, that is, those whose lives are characterized by substantial short falls in much of what makes life go well and whose prospects for a decent life going forward are slim or seriously imperiled. Moreover, an especially critical part of the job of social justice is to identify interlocking patterns of disadvantage that systematically marginalize population groups (Powers and Faden, 2006, 2019).

An example of such an interlocking pattern concerns IPV perpetrated against women. IPV is often perpetrated against women in contexts where women are treated as morally inferior to men (a background social condition), resulting in inequities on all dimensions of wellbeing: impunity for violence perpetrated against them (personal security); exclusion from education opportunities (knowledge and understanding); a lack of respect for women's social and economic contributions (equal respect); social exclusion from extra-familial relationships (personal attachments); and exclusion from political liberties accorded to men (self-determination). The mental health consequences of IPV victimization for women (e.g. the wear and tear on mind and body associated with repeated, overwhelming, activation of the stress response system), in turn further compromise opportunities for women to escape the odds stacked against them. For example, IPV may result in cognitive styles characterized by self-blame and low self-esteem, which may make it harder for women to act out of a position of strength and independence (Kohrt and Bourey, 2016; Greene *et al*., 2019; Tol *et al*., 2019).

Applying a social justice theoretical framework would help further our understanding of, and practice concerning, the complex relationships between interpersonal violence and mental health in at least three important ways:

1. *A social justice framework will focus research efforts on patterns of systematic disadvantage.* With the rapid growth of studies focused on interpersonal violence and mental health, including the rich set of 24 papers in this series, it is easy to get lost in technicalities and relatively narrow debates on the importance of particular variables within specific research traditions. Although the technicalities are critical, a social justice lens can provide a sense of purpose and help maintain focus on the larger issue at stake: to build the knowledge base required to understand how sufficient levels of wellbeing along all its critical elements can be ensured for all. Following Powers and Faden's reasoning, this will require researchers to pay especial attention to identifying particularly pernicious patterns of interlocking social determinants that systematically disadvantage whole population groups, and understand how these patterns may be disrupted.
2. *A social justice lens will compel us to situate the critical issues of concern in our own research and practice disciplines in their broader context.* A unifying framework is needed to avoid fragmentation of research and practice efforts. With relevance to the topic of concern here, seeing interpersonal violence and mental health as two dimensions of a larger set of six elements of wellbeing may help us, for example, to identify currently overlooked relations with other elements of wellbeing (e.g. equal respect), or identify an overlap in social and economic drivers of other elements of wellbeing not previously considered. Moreover, situating our disciplinary-confined concerns in a broader analysis may open up conversations with actors in different practice spaces. Specifically, the emphasis of the above social justice theoretical framework on identifying interlocking patterns of disadvantage may help to stimulate conversations between actors across multiple sectors that are characterized by collaboration and coordination rather than competition. Prioritizing the analysis of how moral concerns interact as components of a shared goal (i.e. wellbeing) is likely more helpful than positioning moral concerns in a zero sum game.
3. *Applying a social justice lens will provide greater conceptual clarity and thus more effectively bridge research and practice*. A social justice framework can put a check on some of the concerns about boundary creep in research that ultimately leads to diffuse intervention and policy recommendations. Defining mental health (as e.g. the WHO does) as an all-encompassing state of wellbeing makes almost all issues of moral importance mental health issues (Powers and Faden, 2006). This conflation presents theoretical challenges, but also has important repercussions for practice. Compounding all moral issues as mental health issues makes it harder to formulate crisp arguments on behalf of mental health, because prioritizing everything may sound similar to decision makers as not prioritizing. Moreover, discussing all dimensions of wellbeing as mental health dimensions runs the risk of inadvertently disempowering advocacy efforts on behalf of various critical social issues as independent moral issues in their own right.

## Re-orienting research: embracing complexity and context in a social justice framework

What does a social justice perspective ask us to do differently with respect to research on interpersonal violence and mental health? Taken together, the findings published across the series’ 24 papers compel us to put complexity and context center stage, rather than to treat them as afterthoughts. A social justice perspective as outlined above encourages a reorientation from the commonplace epidemiologic effort to identify common risk factors (i.e. research focused on the question whether exposure to interpersonal violence is statistically significantly associated with higher levels of mental health concerns) towards a more fine-grained analysis of contextually determined patterns of disadvantage.

A closer look at the findings in this series with regard to IPV in various sub-Saharan African (SSA) settings may help illustrate such a re-orientation in focus. Bajunirwe and colleagues’ cross-sectional study in five SSA sites (*n* = 1415) identified a relationship between IPV victimization and adverse mental health outcomes (i.e. depression and non-alcohol substance abuse) (Bajunirwe *et al*., 2018). Four studies in the series add to our understanding of the social conditions under which IPV is perpetrated in SSA settings. First, an analysis of cross-sectional Demographic and Health Survey data in 14 SSA countries (*n* = 86 024) found a robust relationship between alcohol misuse by a male partner and female reports of IPV victimization. The latter study also suggests a contextual effect, in that living in a country with a higher prevalence of alcohol use was a contributing factor to female reports of IPV victimization independently of her partner's alcohol use (Greene *et al*., 2017). Second, rapid ethnographic formative research in informal settlements in Nairobi, Kenya provides further context on male alcohol misuse in that specific setting: community members perceived that men resort to drinking because of having ‘too much time’, marital conflict, psychosocial issues, and access to alcohol (Schafer and Koyiet, 2018). Third, a cross-sectional study in informal settlements in South Africa found that reports of sexual IPV victimization were more common in women who also report victimization by community violence (e.g. seeing someone being beaten up, seeing a gun in the house) (Malan *et al*., 2018). Fourth, a study focused on more upstream determinants by Stark and colleagues looked at how gender norms are associated with self-esteem. Their cross-sectional study with female refugee adolescents (mainly from Sudan and South Sudan, *n* = 919) found that more equitable gender norms held by peers and community members were associated with higher levels of self-esteem amongst the young women (Stark *et al*., 2018). Collectively, these multi-level dynamics risk negative impacts on the next generation: as noted, a longitudinal study in conflict-affected areas of the Democratic Republic of the Congo found that parental mental health and IPV were predictors of children's wellbeing, including their reports of externalizing behavior and experienced stigma (Glass *et al*., 2018).

From the perspective of the social justice theory outlined above, these findings raise numerous important questions. With regard to alcohol misuse and IPV, for example: What are the background social conditions that explain the relationship between overall prevalence of alcohol use and women's report of IPV victimization? Is overall male alcohol misuse associated with patriarchal gender norms; does the relationship reflect an overall situation of moral degradation in settings of cultural transition, political conflict, or historical trauma? While holding IPV perpetrators accountable for their behavior, what role can mental health interventions focused on reducing male psychological distress and alcohol misuse (e.g. associated with their own experiences of violence victimization) play in preventing or reducing IPV? In addition to addressing men's mental health, which other family-based and community-based interventions may be required to break the intergenerational transmission of violence victimization and subsequent perpetration in low-resource high-adversity contexts?

Regarding the findings on inequitable gender norms and self-esteem, for example: What is the relationship between respect and self-esteem as measured by Stark and colleagues amongst refugee girls in Ethiopia? Is self-esteem a reflection of a cognitive style closely linked to depression (e.g. depression associated with facing continuous overwhelming stressors, including community violence), or better characterized as the result of living in socio-cultural contexts where women are systematically treated as morally inferior? And if both these scenarios are relevant, would a community-based intervention focused on changing inequitable norms related to IPV be sufficient to also address depressive cognitive styles associated with other types of adversities experienced more systematically by women?

Answering such questions will require a stronger emphasis on multi-level longitudinal epidemiological research, as well as a greater investment in multi-disciplinary research efforts. For example, ethnographic research will be critical to understand which connections between interpersonal violence and mental health across multiple social levels (e.g. family, school, community, culture) will be worth investigating in multi-level quantitative epidemiological studies. Qualitative research will also be helpful to understand identified statistical relationships in quantitative studies between more generic variables (such as age and gender) and mental health outcomes. For example, qualitative research would be helpful to identify the particular social processes that resulted in girls and boys experiencing different consequences from family dysfunction in conflict-affected DRC (Glass *et al*., 2018); or why depression symptoms played a different role in the relationship between sexual IPV and suicidality for female and male former child soldiers in Nepal (Bhardwaj *et al*., 2018). Similarly, mixed methods research would be beneficial for understanding the complex patterns at play when intervening on interpersonal violence or mental health. For example, an ethnographic study conducted concurrently with a RCT could focus on how it was possible that a couples-based gender dialogues integrated in an economic intervention may result in a reduction of PTSD symptoms – and why this effect was only observed in women who did not report IPV (Annan *et al*., 2017).

A helpful health research paradigm for the multilevel epidemiological efforts instigated by the questions emerging from this series is the eco-epidemiological paradigm suggested by Susser & Susser. In 1996, Susser and Susser observed that the majority of epidemiology is conducted from within a ‘black box’ paradigm, applying relatively straightforward approaches to causality (i.e. risk factor X causes disease outcome Y). They advocate for a paradigm shift characterized by the image of ‘Chinese boxes’ (i.e. nested systems), and for epidemiology to take advantage of major advances in both social epidemiology and neuropsychological research (Susser and Susser, 1996). Taking advantage of advances in neuropsychological research, for example, may be helpful to answer intervention-focused questions such as whether mental health interventions amongst violence-affected populations result in reduced allostatic load, and whether this in turn mediates future risks for violence and improvements on other dimensions of wellbeing.

In addition, to advance a multi-sectoral, multi-level research agenda, a stronger integration of systems analysis (such as system dynamics modeling) will be helpful to replace more simplistic models of causal analysis (Drigo *et al*., 2012). For example, important intervention questions concern how many (and which) intervention components need to be impacted in a multi-level and interrelated-determined set of disadvantages before a vicious cycle becomes an upward spiral.

In closing, the 24 papers in this series collectively present a rich set of findings. Together, this body of findings suggests intricate bi-directional relationships between interpersonal violence and mental health, situated in particular contexts and varying across the life course. Such processes are challenging to capture with epidemiological research aimed at identifying one-way relationships between interpersonal violence as a risk factor and mental health as the outcome. Together, these 24 studies urge us to embrace complexity and context in research on interpersonal violence and mental health rather than sideline them. This editorial argues that efforts to unravel this complexity would benefit from the application of a social justice theoretical framework. Such application would help to (1) provide a sense of direction to focus on key inequalities that prevent people from obtaining wellbeing with more fine grained analyses of contextually determined processes of disadvantage; (2) situate our own specific research and practice questions in their broader context (hopefully strengthening multi-disciplinary research and multi-sectoral policy and programming); and (3) strengthen conceptual clarity – without losing the forest for the trees. It is likely only through collective efforts – across academic disciplines, governmental departments, and non-governmental agency mandates – that the interlocking patterns of disadvantage that systematically marginalized whole population groups can be effectively identified and remedied.
